# Contaminants fingerprinting in environmental matrices of Radon concentration in groundwater: A baseline study in Alappuzha (Kerala) and the associated health effects

**DOI:** 10.1371/journal.pone.0340896

**Published:** 2026-01-23

**Authors:** Selvam Sekar, Akhila V. Nath, Muthukumar Perumal, Priyadarsi D. Roy, Amin Ullah, Muralitharan Jothimani

**Affiliations:** 1 Department of Geology, V.O. Chidambaram College, Tuticorin, Tamil Nadu, India; 2 Instituto de Geología, Universidad Nacional Autónoma de México (UNAM), Ciudad Universitaria, Ciudad de México, Código Postal, Mexico; 3 Department of Health and Biological Sciences, Abasyn University, Peshawar, Pakistan; 4 Department of Geology, College of Natural and Computational Sciences Arba Minch University, Arba Minch, Ethiopia; University of Peshawar National Centre of Excellence in Geology, PAKISTAN

## Abstract

Accumulation of Radon in groundwater from aquifer lithologies can pose significant health risks. This study investigates its concentration in coastal Alappuzha from the Kerala state in India to assess health risks from ingestion and inhalation in groundwater samples of both the pre-monsoon and post-monsoon seasons. The activity(RAD7detector)in post-monsoon samples remained relatively higher (2.59–66.46 Bq/L; average:20.25 Bq/L) compared to the pre-monsoon (1.63–46.38 Bq/L; average: 13.78 Bq/L), reflecting the effect of precipitation on enhanced Radon contamination. However, the samples presently do not exceed the World Health Organization recommended maximum value (100 Bq/L). Relatively higher average radiation doses for stomach and lungs (6.12–6.20 Svy^-1^) in post-monsoon samples compared to pre-monsoon (4.12–4.22 Svy^-1^) show more exposures in the post-monsoon season. Our estimations of the total effective dose from two different pathways provide valuable baseline data on radon exposure in a coastal region of India, where health risks could increase due to higher precipitation and more frequent heavy rainfall events in the near future. Such conditions may enhance groundwater recharge and promote the downward migration of radon-rich soil gases into aquifers, potentially elevating radon concentrations, particularly in uranium-bearing lithological settings.

## Introduction

At least two of the Sustainable Development Goals aim to improve the water quality by minimizing the effects of contamination, both from the anthropogenic and geogenic sources through different man-made activities such as industrialization, urbanization, and agriculture, and the different effects of global warming. The average global temperature has already increased by 1.1 ºC compared to the pre-industrial period comprising of instrumental registers of 1850–1900 and it has been related to the unprecedented greenhouse gas emissions into the atmosphere [1, 2]. Under the five different scenarios, IPCC has projected an increase between 1.4 and 4.4 ºC of average global temperature by the end of this century and it would be reflected in the enhancements between 10 and 40% of the total annual precipitation as well as associated soil moisture over different parts of India [3,4]. In the conservative SSP2–4.5 scenario, where the global emission of different greenhouse gases would decrease during the second half of 21^st^ century, the IPCC WGI projections show high confidence that the increase of precipitation would be through more frequent events of heavy precipitation and flash floods causing both erosion and flooding in the coastal areas of India.

Even though water makes up to 70–75% of the human body weight, only 0.3% of the world's total water resources are currently appropriate for drinking and daily usage [5, 6, 7]. The contamination of water with high concentration of Radon is considered as one among the different pollution pathways. Radon (^222^Rn) is a radioactive gas naturally present in the groundwater and the variations of ^222^Rn activity in groundwater can span nine orders of magnitude [8, 9]. Different radioactive isotopes disintegrate through the uranium and thorium series in the lithosphere and atmosphere into Radon and this gaseous element easily dissolves into water and hence, it is enriched in groundwater compared to other elements of the uranium series. More than 50% of the radiation exposure to humans comes from ^222^Rn and ^220^Rn, the gaseous daughter products of Th and U, respectively [10].

The exposure of Radon varies as a result of its concentration in soil and water which in turn is influenced by factors such as lithology, soil permeability, moisture content, and the presence of uranium- and thorium-bearing minerals. [11, 12]. Two of the ^222^Rn descendants, ^214^Po and ^218^Po, are emitters and account for more than 90% of the overall radiation dosage [13]. The health risk comes from the inhalation and consumption of these two short-lived decay products. Majority of the radon gas are flushed out from the lungs, but it can harm the DNA of delicate lung tissue and result in cancer. The airborne decay products of radon enter our respiratory system and degrade the lungs in their brief half-lives. Alpha particles, released during these decays, may provide significant amount of energy to the susceptible cells, posing several health risks such as stomach cancer from consuming radon in drinking water and lung cancer from breathing of radon released from the water [14, 15, 16]. The radon-containing water can release radon gas into the air, which can then be inhaled. The solid radon progeny or radon daughter particles, could also be inhaled as they are generally attached to dust or other particulates in the air, enhancing the possibility of lung cancer by harming lung tissue after the inhalation.

The assessment of health uses the concept of effective dosage, which is a measure of the overall radiation dose to the body, taking into account the exposed organs and tissues. The effective dose from radon in water is generally lower compared to the dose from radon in indoor air. Sievert (Sv)measures the biological impact of radiation on human tissues, by considering the amount of radon in water (Bq/L), volume of water consumed, and length of exposure [17]. The exact effective dose from radon in water can vary depending on several factors such as the radon concentration, amount of water consumed, and duration of exposure. For example, the World Health Organization [18] recommended a maximum value of 100 Bq/L. The effective dose typically remains in the range of 0.1–10 millisieverts (mSv) per year for individuals who consume water with radon concentrations at or below the WHO reference level. However, it would be higher for individuals who consume larger amounts of water or are exposed to radon for longer durations. Additionally, the factors such as radon concentration in indoor air, ventilation, and other sources of radiation in the environment can also contribute to the overall effective dose. It’s important to mentioning that different countries have their own guidelines or regulations regarding radon in the drinking water.

Radon is a naturally occurring radioactive gas derived from the decay of uranium in rocks, soil, and groundwater. Although radon itself is not a greenhouse gas, climate change may indirectly influence its environmental behaviour and seasonal variability [19]. Changes in precipitation patterns, soil moisture, and groundwater dynamics induced by climate change can impact radon migration and accumulation. Increased rainfall or flooding can elevate groundwater levels, enhancing radon transport through water sources and potentially increasing its concentration in aquifers and surface waters [20]. Similarly, higher soil moisture during post-monsoon season may impede radon escape to the atmosphere, leading to increased subsurface accumulation and delayed releases [21].Temperature variations also influence indoor and subsurface radon levels. During colder seasons, tightly sealed buildings reduce ventilation, increasing radon accumulation indoors [22]. Conversely, warmer conditions can enhance radon diffusion from the subsurface due to elevated soil temperatures and pressure differentials [23].

Additionally, climate mitigation strategies such as enhanced building insulation can inadvertently increase radon exposure indoors by reducing natural ventilation [24]. These changes underscore the importance of integrated environmental health monitoring under changing climate regimes. While radon is not a contributor to climate change, it serves as a valuable tracer in environmental and geological studies. Radon measurements have been used to monitor permafrost thawing [25], groundwater recharge [26], and even tectonic activity processes that may respond to climate variability. Therefore, understanding the interplay between climate-driven processes and radon dynamics is essential for accurate risk assessment, particularly in regions like Kerala where both hydro geological complexity and climate variability are prominent. While radon is not a primary indicator in climate change research, its measurements can indirectly contribute to understanding environmental processes influenced by climate variability. For example, radon levels are sensitive to geological activity such as seismic disturbances and groundwater level changes, both of which can be affected by climatic shifts [25, 26]. Moreover, radon has been used in permafrost studies to monitor thaw-induced gas emissions [24]. Although radon itself is not a greenhouse gas, its dynamic behaviour under changing temperature and hydrological conditions makes it a useful environmental tracer in the broader context of climate-related studies.

The objectives of the study areai) to analyze the seasonal variation in radon concentrations in groundwater samples collected from the coastal areas of Alappuzha, Kerala, India, during the pre-monsoon and post-monsoon periods and ii) to evaluate the potential health effects associated with radon exposure through ingestion and inhalation pathways, considering the influence of climate change particularly increased precipitation and extreme weather events on radon mobility and distribution. These background values for both the seasons could be useful as a baseline to evaluate the radon contamination in groundwater possibly caused from the effects of global warming.

## Materials and methods

### Sampling

**Fig 1:**
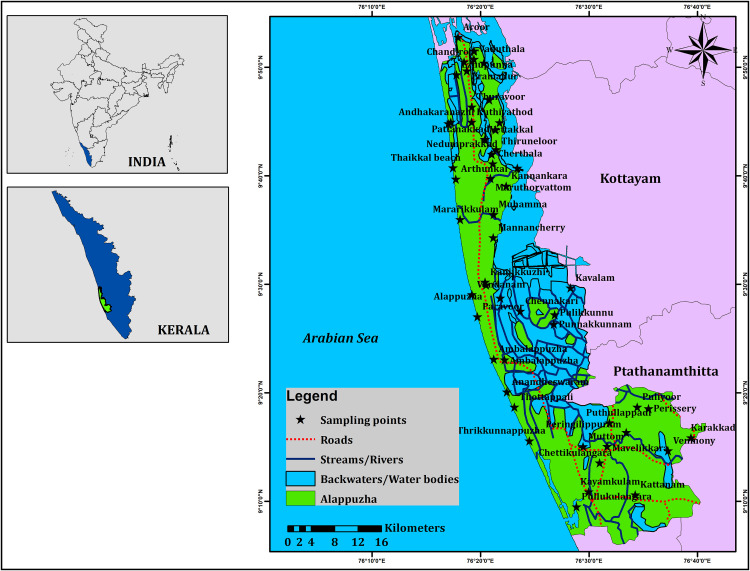
Map showing the location of groundwater samples (pre- and post-monsoon) in coastal areas Alappuzha coastal areas in Kerala state of India.

Alappuzha, also known as Alleppey, is a prominent city located in the southern state of Kerala, India. Located along the Arabian Sea coast, Alappuzha holds a significant geographical importance due to its proximity to water bodies and its role in the state cultural and economic landscape [27]. Geographically, Alappuzha is situated in the southwestern part of India (**Fig 1**), settled between the Laccadive Sea to the west and the Western Ghats Mountain range to the east. It lies at a latitude of approximately 9.4981° N and a longitude of 76.3388° E. This coastal location contributes to its unique climate, which is characterized by high humidity and relatively consistent temperatures throughout the year. The annual precipitation in Alappuzha is a crucial aspect of its climate, as it plays an essential role in sustaining the region’s agriculture and ecosystem. On average, the city receives around 2500 mm of rainfall annually, with the majority of this rainfall occurring between the months of June and November [28]. This period corresponds to the southwest monsoon season in India, when the region experiences a significant influx of moisture-laden winds from the Arabian Sea. The distribution of annual precipitation in Alappuzha follows a distinct pattern. The pre-monsoon period, which includes May, is characterized by relatively lower levels of rainfall, marking the beginning of the pre-monsoon season. This time is crucial for various agricultural activities that depend on irrigation due to the scarcity of natural rainfall. On the other hand, the post-monsoon period, which includes November, is marked by higher levels of precipitation. This is when the region experiences the northeast monsoon, which brings rains from the Bay of Bengal [29]. These rains are important for replenishing water sources and supporting agricultural activities during the following months. The period between June and September is the peak monsoon season, during which Alappuzha receives a substantial portion of its annual rainfall. This heavy rainfall is vital for the region’s paddy cultivation and other agricultural practices. The abundance of water during the monsoon months also contributes to the attractive network of backwaters and lagoons for which Alappuzha is renowned.

### Geology

**Fig 2:**
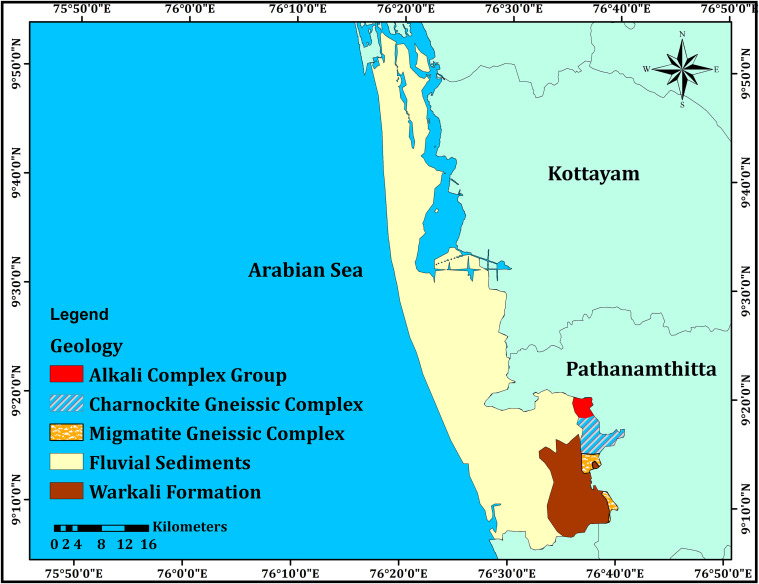
Geology ofAlappuzha coastal areas in Kerala state of India indicating the presence of both crystalline and sedi.mentary formations.

Khondalite group of rocks, with migmatite and charnockites, is the oldest rock type found in the study region. Patches of granite are exposed in the Chengannur area and most of the crystalline rocks are confined to south-eastern side of the study area (**Fig 2**). A small part of the Neogene formation (Miocene age) is exposed at Warkalli in the southern part. Quaternary sediments, comprising Pleistocene to Holocene deposits, are spread throughout the western and central parts of the study area. The Kuttand region is known for Karippadam or carbonized wood formed from the submerged Quaternary forests in the paddy fields. Other quaternary deposits are represented by the paleo-beach deposit as a part of Guruvayur formations, fluvial deposit of the Periyar formation, tidal/mudflat of the Viyyam formation and beach deposit of the Kadappuram formation [30].

The varying lithological units across the study area significantly influence radon distribution. The crystalline rocks, particularly charnockites, migmatites, and granitic intrusions, are known to contain trace amounts of uranium and thorium, serving as potential radon sources. The Warkalli Formation, with its organic-rich sediments and lignite layers, may also contribute to radon release. Seasonal variations in soil moisture, water table levels, and permeability in the Quaternary deposits further affect radon mobility and accumulation, explaining the observed seasonal fluctuations in radon concentrations.

### Hydrogeology

The Alappuzha district, situated along the southwest coast of Kerala, India, features a complex coastal aquifer system shaped by both natural and anthropogenic influences. The region lies within the Vembanad-Kol wetland system and is characterized by a dense network of canals, rivers, lagoons, and backwaters. The aquifer system primarily comprises unconsolidated alluvial and coastal sediments, including fine to medium sand, silt, and clay. Groundwater occurs mainly in shallow phreatic aquifers at depths ranging from 2 to 10 meters below ground level. These aquifers are recharged predominantly through monsoonal rainfall, surface water infiltration, and irrigation return flow. The aquifer system is unconfined to semi-confined in nature, depending on the vertical layering and clay content. Seasonal fluctuations in the groundwater table are significant due to variations in precipitation and tidal effects. The hydraulic conductivity of the aquifer is moderate, supporting domestic and agricultural water demands. However, the proximity to the Arabian Sea renders the aquifer vulnerable to saltwater intrusion, especially in areas of over-extraction. The low topographic gradient and poor natural drainage exacerbate waterlogging and influence aquifer dynamics, particularly during the monsoon season.

### Sampling and analysis

Samples from the Alappuzha coastal areas in Kerala state (India) were collected from groundwater wells (open well, n = 10; bore well, n = 43) during the pre-monsoon month of May (n = 53) and post-monsoon month of November(n = 53) in 2022 for radon analysis, following the protocols of both national and international regulations (**Fig 1**). Each groundwater well is identified by a sample number and location details. All the samples were stored by completely filling 250 ml vial and avoiding any air particles and transported at a temperature of between 4^0^ to 6 ^0^C. The radon concentration was analysed with the automatic radon detector RAD 7 [31,32] in the Environmental Geochemistry Lab, Department of Geology, V.O.Chidambaram College within 24–48 hrs.because of its short half-life (3.8 days).

### Radon concentration

Estimation of radon activity was measured using the Durridge RAD7 radon and thorium detector, similar to other researchers around the world. Its advanced and modern design puts it on level with the most expensive radon measurement tools as it includes a number of unique features such as a rugged carrying case enclosing the instrument, ensuring reliability in the field. Dissolved radon in water was removed in lab by the bubbling air, which was cycled through a closed air-loop through a desiccant tube into the ^222^Rn counting apparatus. The air constantly removes radon until the condition of equilibrium. More than 95% of the radon was eliminated from water by the time the system reaches equilibrium (i.e., within first 5 minutes). The detection accuracy was improved by reducing humidity levels in a desiccant. The ^222^Rn activity was measured for 40 min (4 counting cycles) under equilibrium. The positively charged polonium daughters, ^218^Po (half-life: 3.1 min; alpha energy: 6.00 MeV) and ^214^Po (half-life: 164.3 s; alpha energy: 7.67 MeV), a measure of ^222^Rn concentration in air, were drawn to a high electric field above a silicon semi-conductor detector at ground potential by RAD7.The alpha detector was shielded from dust and charged ions by an air filter near the radon monitor’s entrance. The ions were captured in energy-specific windows that cut out interference and keep background noise at a very low level. ^222^Rn activities were represented here in Bq m^-3^ (disintegration per second per m^3^) with 2σ uncertainty [33]. Additional information regarding the ethical, cultural and scientific considerations specific to inclusivity in global research is included in the Supporting Information (S1 File).

## Results and discussion

### Distribution of radon in groundwater

**Fig 3:**
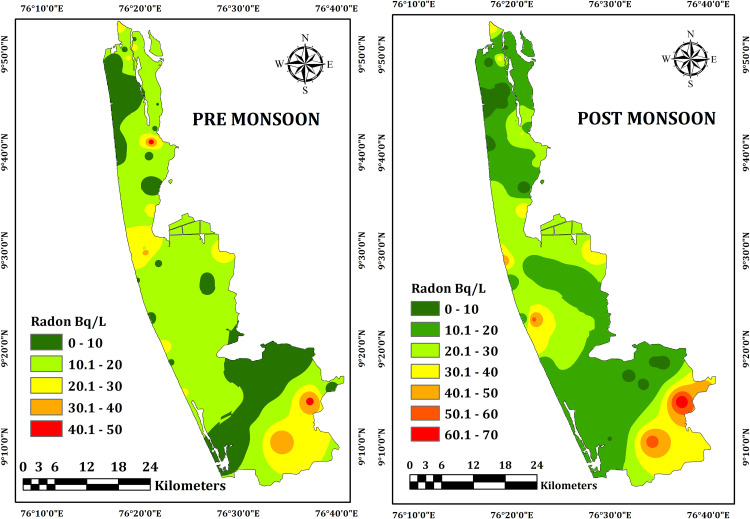
Spatial distribution of radon in groundwater of pre-monsoon (dry season) and post-monsoon (wet season) in the Alappuzha district of Kerala state in India.

Concentrations of ^222^Rn in the groundwater samples of both the seasons are presented in Tables 1 and 2 and Fig 3. In the pre-monsoon season the samples showed a range of activity varying between 1.63 and 46.38 Bq/l (Becquerels per litre), with an average of 13.78 Bq/l. The water samples collected during the post-monsoon, the activity levels slightly increased and ranged from 2.59 Bq/l to 66.46 Bq/l, with an average of 20.25 Bq/l. The activities in water samples from both the seasons are within the moderate range and the radon concentration in none of the seasons exceeded the WHO recommended value of 100 Bq/l. The seasonal change is less substantial but the greater concentrations in the post-monsoon may be due to higher recharge into the aquifer facilitating more radionuclides from the bedrock to dissolve in water. A prolonged dry spell and lowering of the water table previous to the sampling of pre-monsoon samples might have reduced the leaching of radionuclides from bedrock into water. The significance of this observation may be seen from the fact that all water samples during the post-monsoon season and after several months of rainfall in the region showed enhanced radon concentrations [34].

**Table 1:** Values of radon inhalation andingestion, total effective dose and the dose contributions for stomach and lungs in pre-monsoon groundwater samples from 53 locations along the coast of Kerala state in India.

S.No	Location	pH	Radon (Bq/L)	Ingestion Eig(µSv y^-1^)	Inhalation Eih (µSv y^-1^)	Dose Contribution(µSv y^-1^)		Total Effective Dose (µSv y^-1^)
						Stomach	Lungs	
1	Pullukulangara	7.6	6.36	16.25	16.03	1.95	1.92	32.28
2	Kayamkulam	8.4	6.15	15.71	15.50	1.89	1.86	31.21
3	Chettikulangara	8	7.21	18.42	18.17	2.21	2.18	36.59
4	Mavelikkara	7.3	8.56	21.87	21.57	2.62	2.59	43.44
5	Peringilippuram	7.2	5.09	13.00	12.83	1.56	1.54	25.83
6	Perissery	7.3	4.88	12.47	12.30	1.50	1.48	24.77
7	Puliyoor	6.9	5.87	15.00	14.79	1.80	1.78	29.79
8	Puthullappadi	6.7	5.23	13.36	13.18	1.60	1.58	26.54
9	Muttom	7.6	12.4	31.68	31.25	3.80	3.75	62.93
10	Thrikkunnappuzha	7.6	20.5	52.38	51.66	6.29	6.20	104.04
11	Thottappali	8.4	9.26	23.66	23.34	2.84	2.80	46.99
12	Anandheswaram	8.4	24.4	62.34	61.49	7.48	7.38	123.83
13	Ambalappuzha	8.3	8.27	21.13	20.84	2.54	2.501	41.97
14	Ambalappuzha	8.3	12.9	32.96	32.51	3.96	3.90	65.47
15	Pallippuram_malabar	8.3	9.62	24.58	24.24	2.95	2.91	48.82
16	Thaikkattussery	7.6	12.18	31.12	30.69	3.73	3.68	61.81
17	Panavalli	7.6	11.9	30.40	29.99	3.65	3.60	60.39
18	Vaduthala	7.8	25.8	65.92	65.02	7.91	7.80	130.94
19	Arookkutti	8.3	8.49	21.69	21.39	2.60	2.57	43.09
20	Aroor	6.8	23.49	60.02	59.19	7.20	7.10	119.21
21	Chandiroor	7.4	7.35	18.78	18.52	2.25	2.22	37.30
22	Eramallur	7.8	22.71	58.02	57.23	6.96	6.87	115.25
23	Ezhupunna	7.9	3.25	8.30	8.19	1.00	0.98	16.49
24	Kuthiyathod	7.1	4.59	11.73	11.57	1.41	1.39	23.29
25	Thuravoor	8.3	3.86	9.86	9.73	1.18	1.17	19.59
26	Andhakaranazhi	6.8	4.39	11.22	11.06	1.35	1.33	22.28
27	Andhakaranazhi beach	7.8	2.91	7.44	7.33	0.89	0.87	14.77
28	Thaikkal beach	7.6	1.63	4.16	21.57	0.49	0.49	8.27
29	Arthunkal	7.6	8.56	21.87	50.82	2.62	2.58	43.44
30	Pattanakkad	7.8	22.57	57.66	22.07	6.91	6.82	114.54
31	Vettakkal	7.3	8.56	21.87	59.19	2.62	2.58	43.44
32	Nedumprakkad	7.3	20.17	51.53	22.62	6.18	6.09	102.36
33	Pulikkunnu	7.2	8.76	22.38	36.43	2.68	2.64	44.45
34	Kavalam	7.7	23.49	60.01	20.31	7.2	7.13	119.21
35	Punnakkunnam	8	8.98	22.94	75.07	2.75	2.71	45.57
36	Chennakari	7.9	14.46	36.94	23.53	4.43	4.37	73.38
37	Kainakiri	7.6	8.06	20.59	95.45	2.47	2.43	40.94
38	Vandanam	7.2	29.79	76.11	40.92	9.13	9.84	151.18
39	Paravoor	8.3	9.34	23.86	54.73	2.86	2.82	47.45
40	Alappuzha	8.3	37.88	96.78	39.21	11.61	11.4	192.24
41	Kanjikkuzhi	7.6	16.24	41.49	9.19	4.97	4.91	82.41
42	Mannancherry	7.8	21.72	55.49	32.79	6.65	6.56	110.22
43	Mararikkulam	7.2	15.56	39.75	38.85	4.77	4.75	78.96
44	Muhamma	8.3	3.65	9.325	116.87	1.11	1.13	18.52
45	Kannankara	7.6	12.98	33.16	97.87	3.97	3.92	65.87
46	Thanneermukkam	7.8	15.42	39.39	106.64	4.72	4.66	78.25
47	Cherthala	7.2	46.38	118.59	19.85	14.2	14.25	235.37
48	Kattanam	7.2	38.84	99.23	15.77	11.9	11.74	197.11
49	Venmony	7.6	42.32	108.12	43.92	12.9	12.79	214.77
50	Karakkad	7.8	7.88	20.13	15.54	2.41	2.38	39.99
51	Maruthorvattom	7.2	6.26	15.99	21.57	1.91	1.89	31.76
52	Kadakkarapalli	7.1	17.43	44.53	50.82	5.34	5.27	88.45
53	Thiruneloor	6.6	6.17	15.76	22.07	1.89	1.86	31.31

**Table 2:** Values of radon inhalation andingestion, total effective dose and the dose contributions for stomach and lungs in post-monsoon groundwater samples from 53 locations along the coast of Kerala state in India.

S.No	Location	pH	Radon Bq/L	Ingestion Eig(µSv y^-1^)	Inhalation Eih(µSv y^-1^)	Dose Contribution(µSv y^-1^)		Total Effective Dose (µSv y^-1^)
						Stomach	Lungs	
1	Pullukulangara	7.8	10.11	25.83	25.48	3.10	3.06	51.31
2	Kayamkulam	8.8	9.77	24.96	24.62	2.99	2.95	49.58
3	Chettikulangara	8.2	11.46	29.28	28.87	3.51	3.46	58.15
4	Mavelikkara	7.6	13.59	34.72	34.24	4.16	4.19	68.96
5	Peringilippuram	7.4	8.08	20.64	20.36	2.47	2.44	41.66
6	Perissery	7.8	7.75	19.8	19.53	2.37	2.34	39.33
7	Puliyoor	7.6	9.32	23.81	23.48	2.85	2.81	47.29
8	Puthullappadi	7.2	8.31	21.23	20.94	2.54	2.51	42.17
9	Muttom	7.6	19.77	50.51	49.82	6.06	5.97	100.3
10	Thrikkunnappuzha	7.8	18.53	47.34	46.69	5.68	5.63	94.03
11	Thottappali	8.8	14.71	37.58	37.06	4.51	4.44	74.65
12	Anandheswaram	8.8	38.87	99.31	97.95	11.9	11.7	197.2
13	Ambalappuzha	8	13.14	33.57	33.11	4.02	3.97	66.68
14	Ambalappuzha	8.3	52.24	133.4	131.6	16.16	15.7	265.11
15	Pallippuram_malabar	8.4	15.28	39.04	38.55	4.68	4.62	77.54
16	Thaikkattussery	7.9	12.96	33.11	32.65	3.97	3.91	65.77
17	Panavalli	7.9	18.98	48.49	47.82	5.81	5.73	96.32
18	Vaduthala	8.2	8.01	20.46	20.18	2.45	2.42	40.65
19	Arookkutti	8.5	13.48	34.44	33.96	4.13	4.07	68.41
20	Aroor	7.2	37.3	95.3	93.99	11.4	11.27	189.29
21	Chandiroor	7.6	11.68	29.84	29.43	3.58	3.53	59.27
22	Eramallur	7.8	36.06	92.13	90.87	11.56	10.9	183.45
23	Ezhupunna	8.5	5.16	13.18	13.32	1.58	1.56	26.18
24	Kuthiyathod	7.7	7.3	18.65	18.39	2.23	2.27	37.04
25	Thuravoor	8.8	6.13	15.66	15.44	1.87	1.85	31.19
26	Andhakaranazhi	6.8	6.98	17.83	17.58	2.14	2.11	35.42
27	Andhakaranazhi beach	7.9	4.62	11.8	11.64	1.41	1.39	23.44
28	Thaikkal beach	7.6	2.59	6.617	6.526	0.79	0.78	13.14
29	Arthunkal	8.2	13.59	34.72	34.24	4.16	4.19	68.96
30	Pattanakkad	8.2	35.84	91.57	90.31	10.9	10.8	181.8
31	Vettakkal	7.7	13.59	34.72	34.24	4.16	4.19	68.96
32	Nedumprakkad	7.7	32.02	81.81	80.69	9.81	9.68	162.51
33	Pulikkunnu	7.6	13.82	35.31	34.82	4.23	4.17	70.13
34	Kavalam	7.5	37.3	95.3	93.99	11.4	11.27	189.2
35	Punnakkunnam	8.6	14.26	36.43	35.93	4.37	4.31	72.36
36	Chennakari	8.2	14.71	37.58	37.06	4.51	4.44	74.65
37	Kainakiri	8.2	12.8	32.74	32.25	3.92	3.87	64.96
38	Vandanam	7.9	47.3	120.85	119.19	14.5	14.33	240.47
39	Paravoor	8.3	14.83	37.89	37.37	4.54	4.48	75.26
40	Alappuzha	8.3	28.87	73.76	72.75	8.85	8.73	146.51
41	Kanjikkuzhi	7.9	25.78	65.86	64.96	7.94	7.79	130.83
42	Mannancherry	7.8	34.48	88.09	86.88	10.5	10.42	174.98
43	Mararikkulam	7.6	24.71	63.13	62.26	7.57	7.47	125.43
44	Muhamma	8.8	5.8	14.81	14.61	1.77	1.75	29.43
45	Kannankara	7.9	20.61	52.65	51.93	6.31	6.23	104.59
46	Thanneermukkam	7.9	24.49	62.57	61.71	7.58	7.45	124.28
47	Cherthala	7.8	26	66.43	65.52	7.97	7.86	131.95
48	Kattanam	7.8	54.04	138.72	136.18	16.56	16.34	274.25
49	Venmony	7.9	66.46	169.85	167.47	20.37	20.97	337.28
50	Karakkad	8.5	42.52	108.63	107.15	13.36	12.85	215.78
51	Maruthorvattom	8	9.94	25.39	25.04	3.04	3.58	50.44
52	Kadakkarapalli	7.9	27.67	70.69	69.72	8.48	8.36	140.42
53	Thiruneloor	6.8	9.8	25.03	24.69	3.46	2.96	49.73

Radon concentration remained lower in the open well compared to the bore wells, in line with observations reported by [35] and [36]. For examples, it ranged between 3.25–25.8 Bq/l in open wells and 1.63–46.38 Bq/l in borewells for the pre-monsoon season. Similarly, the activities in open wells varied between 5.16–20.61 Bq/l and in borewells between 2.59–66.46 Bq/l in the post-monsoon samples. The interaction with aquifer lithology might have influenced the different activities of open and bore wells. The open wells have bigger surface area and higher ^222^Rn escape rate compared to the borewells and hence there was less radon concentration in the open well samples of both the seasons. In case of bore wells, the radionuclides from the bedrock were leached into the water at a faster rate and the smaller surface area led to lesser escape rate. The concentration of radon also remained higher in samples collected from the deeper wells. For examples, the sample of a 15 m deep borewell showed radon activity of 66.46 Bq/l and it remained 14.71 Bq/l in the samples collected from a relatively shallower (5 m deep) bore well.

### Hydrochemical facies

**Fig 4:**
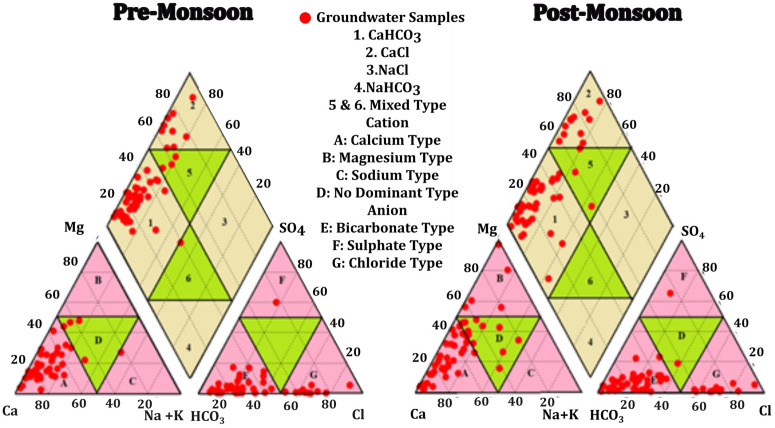
Piper diagram illustrating the hydrochemical facies of groundwater samples from the Alappuzha coastal aquifer.

Hydrochemical facies depicts the chemical attributes of groundwater and furthermore it can delineate the origin and distribution of chemicals within the groundwater [37,38]. Piper diagram [39] is the most common method used to establish hydrochemical facies in the field of hydrogeology [40, 41, 42]. Piper diagram **(Fig 4)** generated for the groundwater in the study reveals that majority of the samples are calcium bicarbonate type (Ca-Mg-HCO_3_) in both pre monsoon (62%) and post monsoon (69%) seasons. The Ca-Mg-HCO_3_ indicate that the water has temporary hardness. This indicates the influence of weathering of bed rock in the study area [43]. 25% of the pre monsoon and post monsoon samples are calcium chloride water type. In the Piper diagram, 11% of pre-monsoon samples and 4% of post-monsoon samples fall in the no dominant ion zone, with the left and right triangles representing cations and anions in the water, respectively. In pre monsoon, evaluation of the triangle that represent cations reveals that 88% of the samples represent calcium type water, 2% are sodium type and 9% of the water samples has no dominant cations. In the anion triangle, 71% of the samples are bicarbonate type and 26% of the water samples are distributed in the chloride type. In post monsoon, 66% are calcium type, 10% are magnesium type and 22% of the samples has no dominant cations present. In the case of anion, 72% of the samples are bicarbonate type, 2% of the samples are sulphate type, 22% of them are chloride type and 2% of the samples has no dominant anion.

### Health effects from radon

The health effects on population of this region have been assessed considering the following radiological effects from radon in groundwater. Dosage from inhaling and ingesting were considered separately for estimating the ^222^Rn dose. The amount of daily water consumption of a person has a significant impact on the effective dosage in case of ingestion. Similarly, the escape of ²²²Rn gas from drinking water into indoor air during household activities such as bathing, cooking, or cleaning can increase the risk of lung cancer due to prolonged inhalation exposure.The annual effective doses from ingestion (D_ing_) and inhalation (D_inh_) of ^222^Rn were determined after considering the parameters defined in [44] as follows:


$$D_{ing}\left(\mu Sv/y\right)=C_{Rnw}\;\times \;10^{-3}\times C_{W}\times EDC$$ (1)

where D_ing_ is the effective dose for ingestion.

C_w_ is the weighed estimate of water consumption (2 L/d).

C_Rnw_ is the ^222^Rn concentration in water (Bq/l).

EDC is the effective dose coefficient for ingestion (3.5nSv/Bq).


$$D_{inh}(\mu Sv/y)=\;C_{Rnw}\times R_{aw}\times I\times F\times DCF$$ (2)

where D_inh_ is the effective dose for inhalation.

C_Rnw_ is the ^222^Rn concentration in water (Bq/l).

R_aw_ is the ^222^Rn in air to ^222^Rn in water ratio (10^−4^).

F is the equilibrium factor between ^222^Rn and its progenies (0.4).

I is the average indoor occupancy time per individual (7000 h/y)and

DCF is the dose conversion factor for ^222^Rn exposure [9 nSv(Bqhm^-3^)^-1^].

Additionally, the ingestion-related dose contribution to lungs and stomach were also estimated by multiplying the ingestion and inhalation doses with the tissue weighting factor of lungs and stomach (0.12) [45, 46, 47].

**Fig 5:**
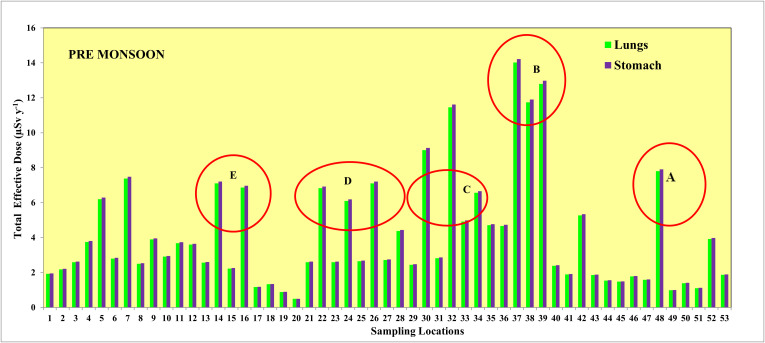
Total effective dose in stomach and lungs from the ingestion of pre-monsoon (dry season) groundwater of 53 wells in the Alappuzha district of Kerala state in India.

**Fig 6:**
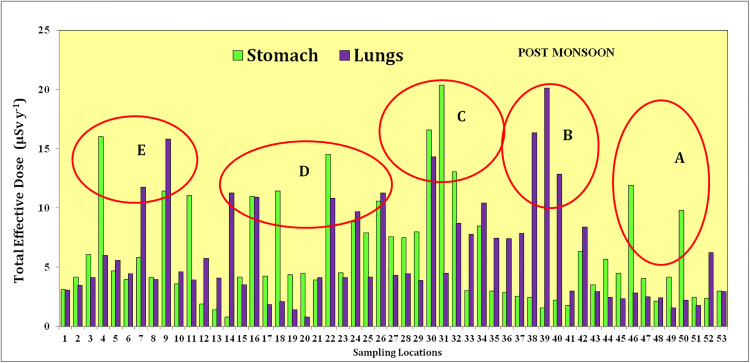
Total effective dose in stomach and lungs from the ingestion of post-monsoon (wet season) groundwater of 53 wells in the Alappuzha district of Kerala state in India.

In the pre-monsoon samples, the effective dose for ingestion varied from 4.16 to 118.5 Svy^-1^(average:35.2 Svy^-1^), and the effective dose for inhalation ranged from 4.1 to 116.8 Svy^-1^(average:34.74 Svy^-1^) (Table 1). The average radiation dose for the stomach was 4.22 Svy^-1^, and it showed a comparable value for the lungs (4.16 Svy^-1^). The average annual total effective dosage for adults remained 69.96 Svy^-1^(**Fig 5**). In the post-monsoon samples, the estimated level of exposure from ingestion ranged from 6.16 to 169.8 Svy^-1^(average: 51.74 Svy^-1^) and the exposure from inhalation varied between 6.5 and 167.47 Svy^-1^(average of 51.03 Svy^-1^) (Table 2). The average radiation doses for stomach (6.20 Svy^-1^) and lungs (6.12 Svy^-1^) again showed comparable values. The average annual effective dosage for an adult from the post-monsoon samples increased to almost twice compared to the pre-monsoon (i.e., 102.78 Svy^-1^, **Fig 6**). Even though the differences remained less significant, the exposures through both ingestion and inhalation (for stomach and lungs) were higher from the post-monsoon season groundwater samples compared to the pre-monsoon season groundwater samples.

Comparison with the literature provides evaluation of the findings from the current study with other studies within India and across the world with similar and different analytical equipment (Table 3). In the group of data with similar methodology (i.e., Radon emanometry using RAD 7), the results from Punjab (0.90–5.10 **Bq/l)** and Rajasthan (1.60–5.40 Bq**/l)** in India shows higher values compared to this study [49, 50]. About 84.9% (n = 45) of the samples of pre-monsoon and 96.2% (n = 51) samples of post-monsoon in the Alappuzha district of Kerala state showed higher activity compared the other two regions such as Rajasthan and Punjab of India using RAD 7 emanometry technique. In the group of data with different methodologies (i.e., Radon emanometry using Alpha Guard and Radon emanometry using Bubbler Method), the activity of Radon in groundwater from Tumkur and Ramnagar District (Karnataka state) and Jaduguda (Jharkhand) remained more than 5-fold higher compared to the coastal region of Kerala state [48, 51].Similarly, the activities of both the seasons remained much higher compared to the studies in Turkey and Pakistan [53, 54].

**Table 3:** Comparison of radon concentration in present study with reported values from other regions using similar and different methodologies.

S.No	Location	Radon concentration (Bq/L)	Method	References
1	Alappuzha District, Kerala India	1.63-66.46	Radon emanometry using RAD 7	Present Study
2	Tumkur and Ramnagar District, India	2.96 - 299.06	Radon emanometry using Bubbler Method	[48]
3	Mandya region, India	6.44 - 44.83	Radon emanometry using Bubbler Method	[36]
4	Rajasthan, India	1.60-5.40	Radon emanometry using RAD 7	[49]
5	Punjab,India	0.90-5.10	Radon emanometry using RAD 7	[50]
6	Mysore Taluk, India	0.50 - 6.43	Radon emanometry using Bubbler Method	[35]
7	Jaduguda, Jharkhand,India	7.50-389.60	Radon emanometry using Alpha Guard	[51]
8	Thirthahalli taluk, Karnataka, India	0.37-87.02	Radon emanometry using SRM	[52]
9	Turkey	0.31- 13.14	Radon emanometry using Alpha Guard	[53]
10	Pakistan	2.00-7.90	Gamma Spectrometry	[54]

**Fig 7:**
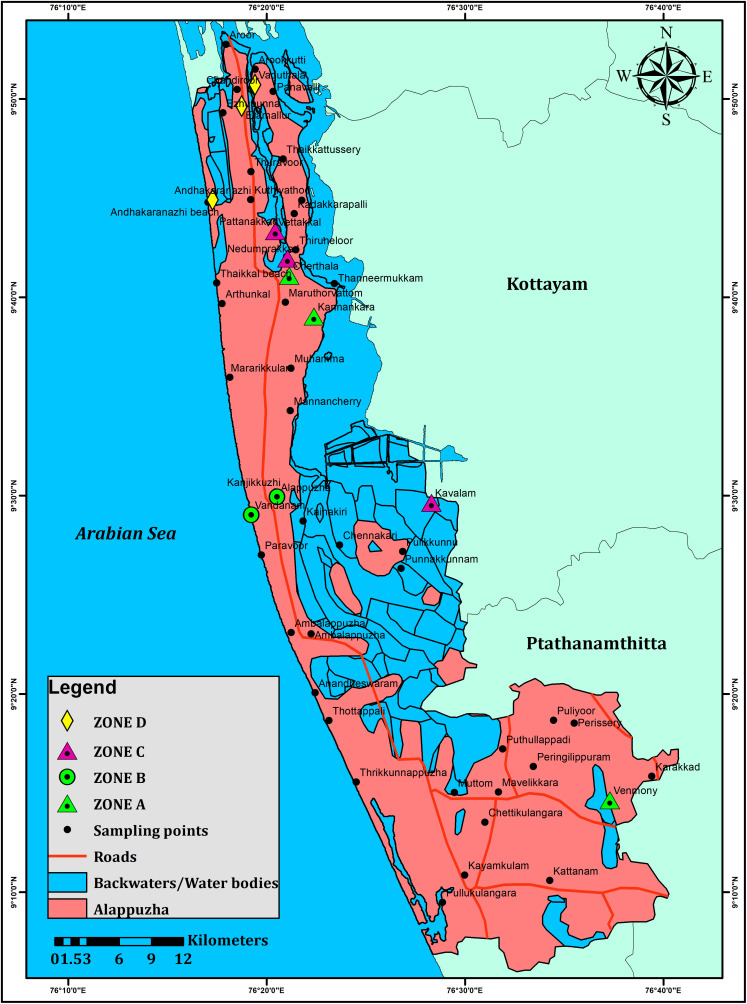
Spatial representation of different zones of Radon.

The total effective dose on stomach and lungs from the exposures to groundwater from both the seasons demarcated five zones such as A,B,C,D and E with higher values (Figs 5–7).

#### Zone A.

It included the sampling locations between 47–50 for both the seasons and showed the highest total effective dose values. Total effective dose on stomach and lungs of this zone ranged up to 14 µSvy^-1^ in the pre-monsoon season and increased to 20 µSvy^-1^ in the post-monsoon season. The geology might have played an important role as this zone has underlying crystalline rocks composed of migmatites and charnockites. The occurrence of uranium in these lithologies as well as the overlying soil might have produced radon. The leaching of ^222^Rn into the groundwater from the bed rocks might have enhanced the radon activity in both the seasons with the activity during the post-monsoon season remaining much higher compared to the pre-monsoon due to more radionuclide leaching into the groundwater through the process of aquifer recharge.

#### Zone B.

This zone included locations between 38 and 42 with the total effective dose on stomach and lungs from pre-monsoon samples (up to 12 µSvy^-1^) and post-monsoon samples (up to 15 µSvy^-1^) remaining comparable. The source of radon is from the hydromorphic soils that releases radon from the decay of radium bearing minerals such as uranium and thorium minerals. These minerals can be found in various types of rocks and soils, including granite, shale, and phosphate-rich rocks. In both the seasons, the comparative amounts of radon atoms produced by radioactive decay were released into soil pores and subsequently to the atmosphere rather than leaching into the groundwater.

#### Zone C.

The locations of 10,12, 30, 32 and 34 represented this zone. Total effective dose effect on stomach and lungs rangedup to10 µSvy^-1^ in the pre-monsoon samples and slightly increased up to 15 µSvy^-1^ in the post-monsoon samples. Considering the geology, the sources of radon for this zone are the alluvial soils and lateritic soils. The groundwater from this zone showed the generally less effect on the human health from inhalation and ingestion Rest of the samples showed small amount of radon and the geology is represented by presence of carbonaceous clay.

#### Zone D.

The sampling locations of 18, 20 and 22 having the Total effective dose effect on Lungs and Stomach ranges up to 8 μSvy^-1^ in premonsoon season. Here the sources of radon are from the alluvial soils. In Post Monsoon, the Total effective dose effect on Lungs and Stomach range up to 12 μSvy^-1^.

#### Zone E.

In premonsoon, the sampling locations of 10 and 12 having the Total effective dose effect on Lungs and Stomach ranges up to 10 μSvy^-1^. Here the sources of radon are from the alluvial soils. In post monsoon, the sampling locations of 12 and 14 Total effective dose effect on Lungs and Stomach range up to 15 μSvy^-1^.

Heavy or sustained rainfall increases groundwater recharge, which can enhance contact between water and radon-producing minerals (like uranium-rich granites or certain metamorphic rocks) in the aquifer. This can increase radon dissolution into groundwater. Rainfall can push radon-rich soil gas deeper into the saturated zone, allowing it to dissolve in groundwater. Sometimes, increased recharge can *dilute* radon if the water passes quickly through the system. However, in aquifers with high uranium content, the opposite can happen rapid infiltration can mobilize fresh radon into groundwater before it decays. Many studies in India and elsewhere show that radon levels in groundwater often vary seasonally, with peaks during or soon after the monsoon due to these recharge and flushing processes. Even through the maximum values of the radon concentration in groundwater of both the seasons did not exceed the WHO recommended value, some key points regarding the human health effects from radon have been listed here:

1. **Lung cancer:** Radon is thought to be the major cause of lung cancer in many parts of the world, and it is followed by smoking [55]
2. **More cancer risk for smokers**: Lung cancer risk can be considerably increased by smoking cigarettes and being exposed to radon together [56]
3. **Inhalation of radon progeny**: Radon gas itself is relatively inert and does not directly cause significant health effects. It, however, produces solid particles known as radon progeny or radon daughters through radioactive decay. The risk of lung cancer can be raised by the alpha particles that the radon progeny generates [57].
4. **Contribution of radon in water**: Radon in drinking water contributes to the overall radon exposure, but the contribution is generally smaller compared to radon in indoor air. The risk depends on the amount of radon in water, as well as how often and for how long one is exposed [58].
5. **Other health effects:** There may be a connection between radon exposure and respiratory conditions such respiratory tract infections and chronic obstructive pulmonary disease (COPD) [59]. However, the enhancement of proper ventilation and reduction in radon levels in the indoor air can minimize overall radon exposure.

## Conclusion

The present study provided important baseline data about radon levels in groundwater from the pre-monsoon season and post-monsoon season in the Alappuzha coastal aquifers of Kerala, India and conducted a comprehensive health risk assessment through total effective dose calculation through two different pathways on stomach and lungs. The results demonstrated variations in radon concentrations (always below the WHO recommended values) in the groundwater sources (borewell and open well) within the study area as well as the seasonal changes. The findings of this study highlight the importance of radon exposure in coastal aquifers to safeguard public health considering the fact that the radon activity as well as potential health risk remained higher in groundwater of the post-monsoon season compared to the pre-monsoon. It showed the effect of higher recharge into the aquifer after several months of precipitation facilitating more radionuclides from the bedrock. The variations in radon concentrations among different groundwater sources emphasized the need for targeted interventions and mitigation strategies. Implementation of effective water treatment techniques and guidelines for safe water consumption can significantly minimize the potential health impacts of radon exposure as the climate change is projected to cause the events of heavy precipitation as well as flash flooding more frequently in the coastal areas of India leading to more radon contamination in groundwater.

## Supporting information

S1 File Inclusivity in global research. (DOCX)
